# Improving Multicolor Colocalization in Single-Vesicle Flow Cytometry with Vesicle Transit Time

**DOI:** 10.1021/acs.analchem.3c01197

**Published:** 2023-07-05

**Authors:** Luca A. Andronico, Seung-Ryoung Jung, Bryant S. Fujimoto, Daniel T. Chiu

**Affiliations:** † Department of Chemistry, University of Washington, Seattle, Washington 98195, United States; ‡ Department of Women’s and Children’s Health (KBH), Karolinska Institutet, Solna 17177, Sweden

## Abstract

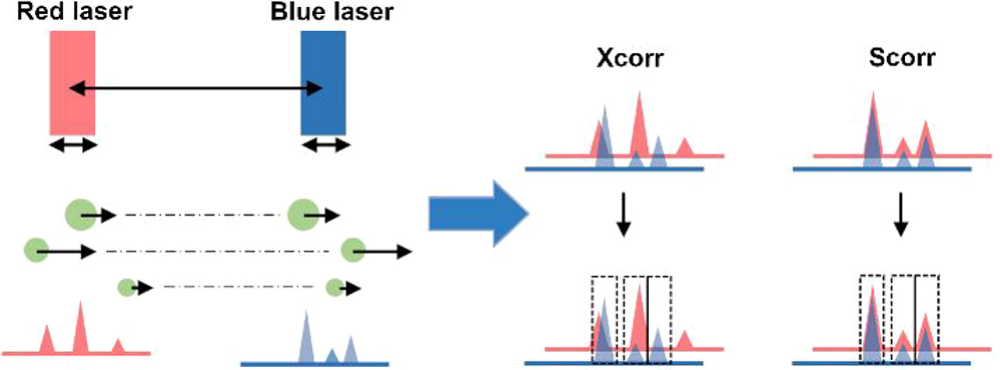

Immunophenotyping of vesicles, such as extracellular vesicles (EVs), is essential to understanding their origin and biological role. We previously described a custom-built flow analyzer that utilizes a gravity-driven flow, high numerical aperture objective, and micrometer-sized flow channels to reach the sensitivity needed for fast multidimensional analysis of the surface proteins of EVs, even down to the smallest EVs (e.g., ∼30–40 nm). It is difficult to flow focus small EVs, and thus, the transiting EVs exhibit a distribution in particle velocities due to the laminar flow. This distribution of vesicle velocities leads to potentially incorrect results when immunophenotyping nanometer-sized vesicles using cross-correlation analysis (Xcorr), as the order of appearance of the vesicles might not be the same at different spatially offset laser excitation regions. Here, we describe an alternative cross-correlation analysis strategy (Scorr), which uses information on particle transit time across the laser excitation beam width to improve multicolor colocalization in single-vesicle immunoprofiling. We tested the performance of the algorithm for colocalization analysis of multicolor nanobeads and EVs experimentally and via simulations and found that Scorr improved both the efficiency and accuracy of colocalization versus Xcorr. As shown from Monte Carlo simulations, Scorr provided an ∼1.2–4.7-fold increase in the number of colocalized peaks and ensured negligible colocalization of peaks. In silico results were in good agreement with experimental data, which showed an increase in colocalized peaks of ∼1.3–2.5-fold and ∼1.2–2-fold for multicolor beads and EVs, respectively.

## Introduction

Flow cytometry remains one of the most popular techniques in the analysis of single cells with countless applications in basic and clinical research. Flow cytometry is used to study cell morphology (e.g., size, granularity) or functional state (e.g., cell proliferation, apoptosis) in health and disease, by examining protein and/or gene expression.^1^ The reason for the success of flow cytometry lies in its capacity to deliver high-throughput multiparametric (up to 43 parameters per cell)^2,3^ analysis with single-cell sensitivity.

The multidimensionality of flow cytometry analysis has led to the emerging field of computational cytometry,^4^ which focuses on data-driven algorithms^5^ to find new automated and unsupervised methods for cell clustering, rare population discovery, biomarker identification, and cell developmental modeling.^6^ Historically limited to the analysis of cells, flow cytometry has been recently used also to investigate cell-derived particles with sizes below one micrometer, such as viruses (Vs)^7^ and extracellular vesicles (EVs).^8−10^

Recently, we described a flow system capable of analyzing EVs with sizes from 30 to 300 nm^11,12^ and showed that new constraints arise when sizing and immunophenotyping such vesicles in our system, due to the velocity distribution of particles within the micrometer-scale flow channel. Here, we propose a new method (Scorr) for colocalization analysis of vesicles and particles, which uses information on single-vesicle/particle transit time across the laser excitation beam width and overcomes limitations of the classical cross-correlation analysis approach (Xcorr). We compared the performance of the two strategies for colocalization via Monte Carlo simulations, then applied them to analyzing 100 nm multicolor beads and exosomes (a subset of EVs)^13^ in a micrometer-size channel. The Scorr approach led to a notable increase in the efficiency and accuracy of multicolor peak correlation, which is crucial during EV immunoprofiling. Therefore, our method for colocalization may allow for a better understanding of the intricate complexity of many biologically relevant vesicles and particles, such as EVs,^14^ Vs,^15^ synaptic vesicles (SVs),^16^ etc.

## Experimental Section

### Sample Preparation

Multicolor beads (100 nm average diameter) excitable by 488, 561, and 640 and 640 nm lasers were purchased from ThermoFisher. The stock solution of beads was diluted 1:1000, 1:100, or 1:10 with Milli-Q water to prepare a low-, medium-, or high-density particles solution, respectively. Exosomes were isolated from seminal fluid according to the literature^11^ and stained with a membrane dye—Di-8-ANEPPS—and an Alexa Fluor 647-tagged (AF647) Anti-CD9 antibody for two-color colocalization experiments, following a previously established protocol.^12^

### Monte Carlo Simulations and Scorr

Scorr and the algorithm to generate simulated flow data were written in MATLAB. For simulations, the following parameters were chosen to resemble previous experimental data obtained with the same flow analyzer:^11^ (i) a parabolic profile for the particle velocity distribution (according to the Poiseuille equation, with a maximum speed of 3 μm/ms, and a channel diameter of 2 μm), (ii) laser beam width, 1 μm, and (iii) distance between lasers, 15 μm. We analyzed three different scenarios of particle densities: low (5 particles/s), medium (35 particles/s), and high (252 particles/s) density, which resembled the experimental conditions of multicolor beads (see below). The intensity ratio of bright-to-dim peaks was set to 3, and the fraction of bright-to-dim peaks was set to either 5% or 50%. Particle locations were generated according to experimental data from multicolor beads, and time spent crossing each laser (*PT*) was randomized to better represent the real scenario of particles flowing within the 2 μm wide channel. See pp 2–6 in the SI for an in-depth description of the Scorr algorithm and workflow, Monte Carlo simulations, and derivation of eqs 2 and 3.

### Flow Data Acquisition and Analysis

The optical setup and protocol for microchannel fabrication have been described previously.^11^ For colocalization analysis, 10 μL of the bead solution (1:1000, 1:100, or 1:10) or exosomes (prestained with Di-8-ANEPPS and AF647-tagged Anti-CD9) was loaded into the PDMS microchip inlet reservoir using gravity-driven flow, and the flow trajectories in the blue, green, and red channels were collected for ∼2 min by operating the avalanche photodiode system at high sampling rate (10 kHz for vesicle speed estimation).^11^ Colocalization analysis was performed by using the Scorr and Xcorr algorithms in MATLAB.

## Results and Discussion

### Transit Time-Dependent Single-Particle Colocalization

**Figure 1:**
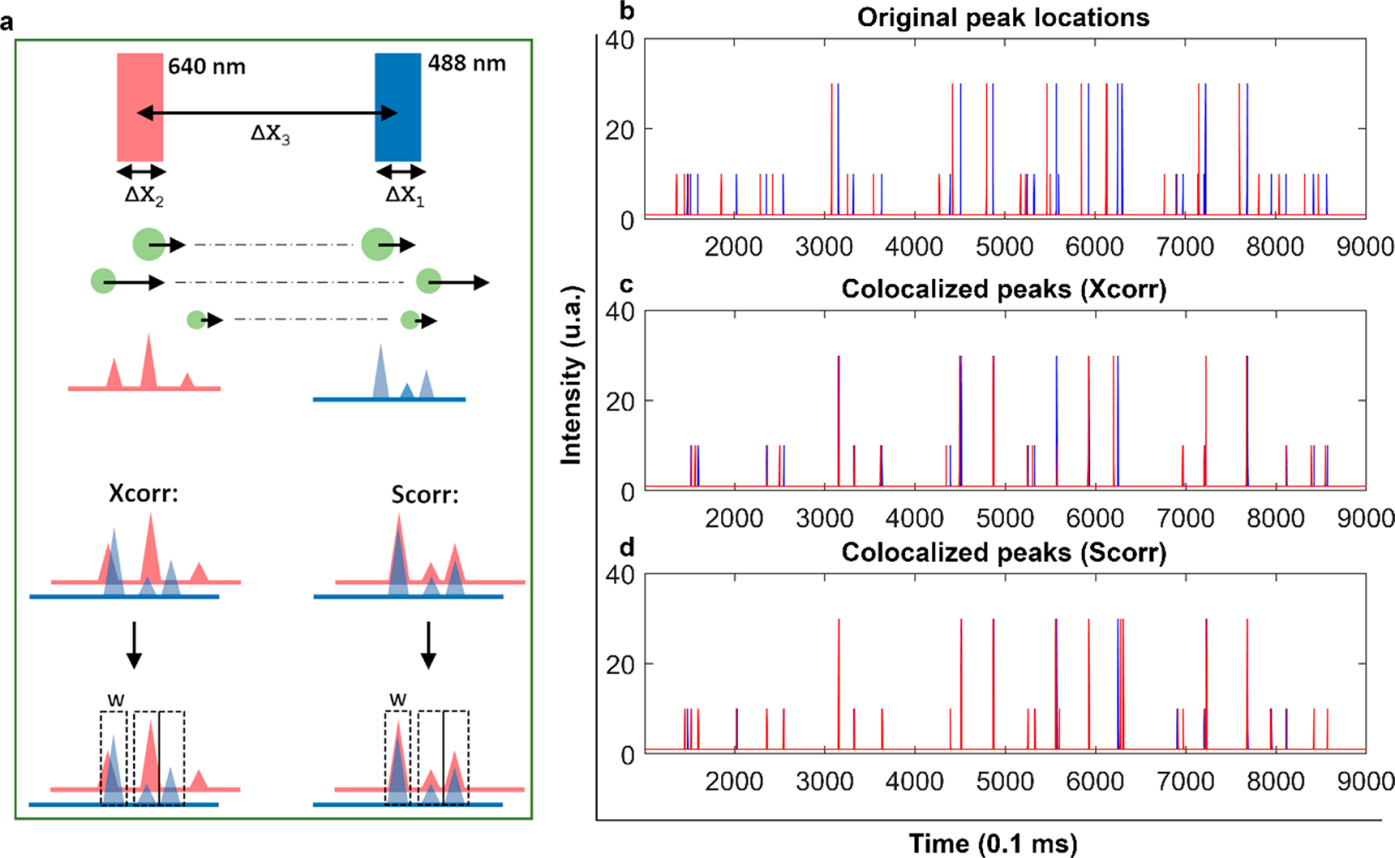
(a) Schematic of the Scorr approach. Circles represent vesicles of different sizes flowing at different speeds (black arrows) across the red and blue lasers. Each peak height is matched with a corresponding vesicle size. Scorr properly colocalizes each peak via single vesicle shifting. (b) Overlap between simulated blue and red trajectories (medium particle density) before shifting for colocalization. (c, d) Overlap between blue and red trajectories (medium particle density) after shifting using Xcorr (c) or Scorr (d).

If multicolor particles such as EVs labeled with different fluorophore antibody conjugates flow through a channel in a thin streamline at the same speed, then they will generate trajectories (one per excitation laser) that are perfect replicas of one another but are displaced in time, with a constant time lag that is directly proportional to the laser-to-laser distance. However, it can be difficult to flow focus EVs and small nanoparticles,^17,18^ and particles will have different flow velocities distributed according to Poiseuille’s law.^19^ As a consequence, the order of appearance of the vesicles might not be the same at different lasers (Figure 1a). Typical approaches for peak colocalization rely on the cross-correlation function (Xcorr, eq 1) to find the delay time (*k*) between two trajectories (*x* and *y*) and to define a cutoff time window (*W*) within which two intensity signals are considered to derive from the same particle.
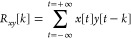
1However, Xcorr operates on each trajectory as a whole; therefore, *k* (which maximizes *R*_*xy*_) represents an average value and does not account for the velocity distribution. This has two main consequences: (i) Since *W* is defined according to *k*, particles moving slower than the average will be discarded (not colocalized), and those moving faster might be double colocalized. (ii) Two particles with different speeds might switch their order of appearance at different interrogation volumes, leading to incorrect colocalization. Furthermore, because of the summation in eq 1, *k* also depends on the peak intensities. Therefore, a small subpopulation of bright particles (e.g., noncolocalized particles, or worse, dye aggregates) might change the lag time, thus affecting the colocalization. To overcome these limitations of Xcorr, we describe a method to perform single-particle colocalization (Scorr), which relies on information about the time spent by the particle to cross the *i*th laser (*PT*_*i*_, estimated during particle detection, see DI for complete derivation).^11^ Briefly, if we consider a two-laser system with excitation at 488 and 640 nm and particles flowing from the red to the blue (Figure 1a), each peak from the red trajectory undergoes two consecutive time shifts (eq 2 and 3, see SI, pp 4–5) to overlap with the respective peak in the blue trajectory (Figure 1b). Equation 2 gives a first approximate shift (coarse shift) by assuming that the particle speed remains constant between the lasers, whereas eq 3 improves the shift (fine shift) by considering an average velocity.
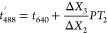
2
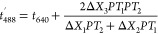
3
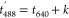
4Our method differs from the Xcorr approach (eq 4) because it applies a particle-specific shift (second term in the right side of eqs 2 and 3), based on information about the blue (Δ*X*_1_) and red (Δ*X*_2_) laser widths, the distance between them (Δ*X*_3_), and the times required to cover those lengths (*PT*_1_, *PT*_2_, and *PT*_3_, see SI, pp 5). In eqs 2–4, *t*′_488_ is the theoretical time at which the *i*th particle, crossing the red laser at *t* = *t*_640_, would appear in the blue trajectory. After the coarse shift, the quantity Δ*T* = |*t*_488_ – *t*′_488_| is calculated according to eq 2, and only those peaks with Δ*T* ≤ *W*_1_ (cutoff window) will be selected to undergo fine shifting. After the fine shift, the quantity Δ*T* is recalculated, this time according to eq 3, and only those peaks with Δ*T* ≤ *W*_2_ will be considered colocalized. The first cutoff window (*W*_1_) depends on the particles’ densities; at higher densities, *W*_1_ should decrease to avoid wrong selection of peaks and respective *PT*_1_ values being used in eq 3—whereas *W*_2_ allows one to remove those peaks which undergo wrong shifting, due to unpredictable changes in the particle’s speed (e.g., upon interaction with the channel walls, etc.). In case of double colocalization, the algorithm identifies the true colocalized peaks as those which have the minimum Δ*T* or those whose *PT* changes the least between lasers (in case of multiple Δ*T* minima (Figure S2)). This two-step shifting approach greatly improves the colocalization accuracy, as illustrated in the simulation results shown in Figure 1b–d and described below.

### Monte Carlo Simulation of Two-Color Colocalization

**Figure 2:**
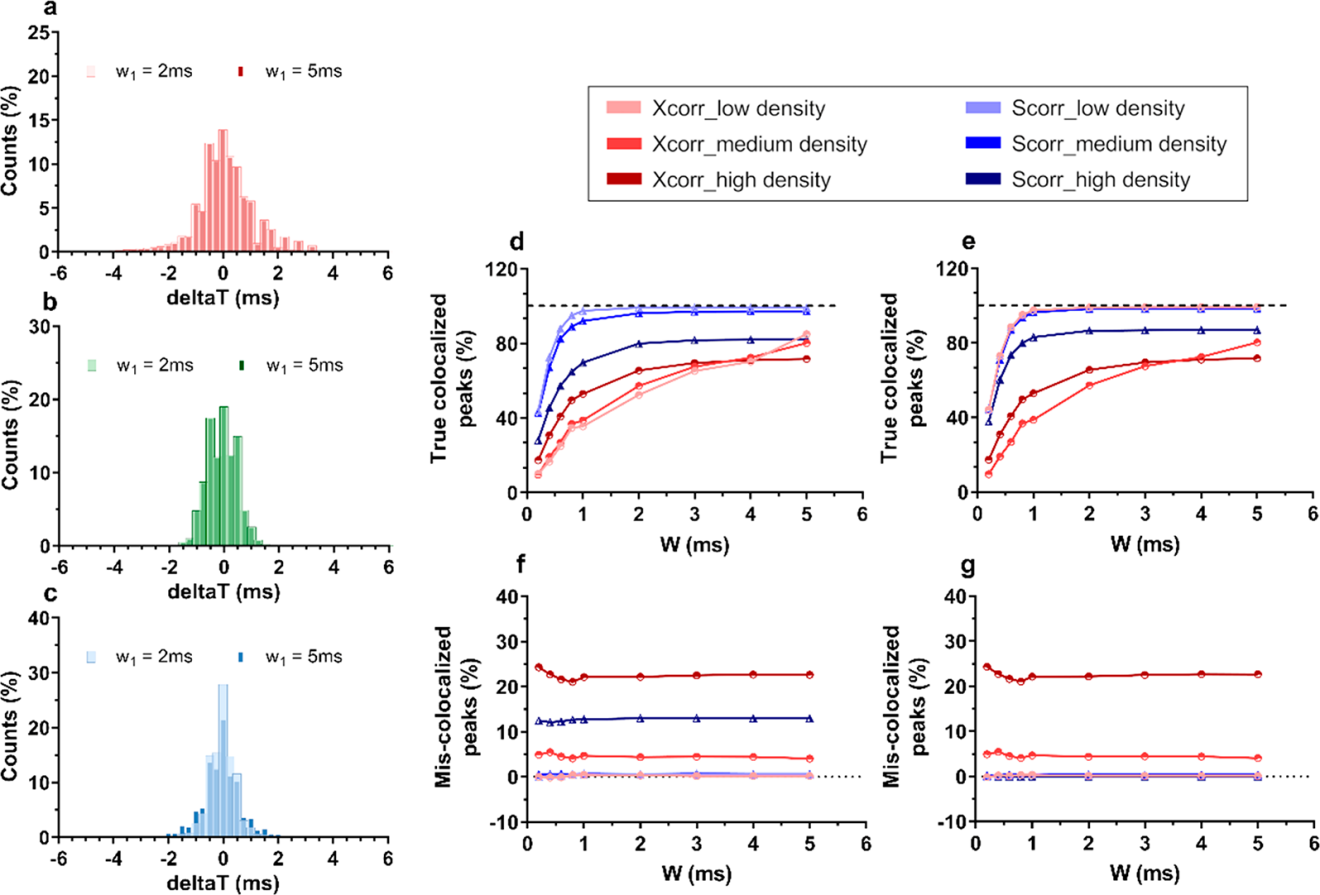
(a–c) Distribution of Δ*T* |*t*′_488_ – *t*_488_| of colocalized peaks, with *t*′_488_ calculated according to eqs 3 and 4 obtained from simulated data (high-density peaks, 1:1 dim–bright mixture), setting *W*_1_ equal to either 2 ms (wide bars) or 5 ms (thin bars) and *W*_2_ = 4 ms. Δ*T* values are obtained after shifting using (a) Xcorr, (b) Scorr using only coarse shift, or (c) Scorr with both coarse and fine shifts. (d, e) Percentage of true colocalized peaks as a function of *W*_2_, at different particles densities (low, medium, high = 5, 35, 252 particles/s, respectively), setting *W*_1_ equal to either 5 ms (d) or 2 ms (e). Dashed lines in panels d and e indicate 100% colocalization. (f, g) Percentage of mis-colocalized peaks (dim peaks with brighter ones) as a function of *W*_2_ at different particles densities, setting *W*_1_ equal to either 5 ms (f) or 2 ms (g). The color code in the legend refers to plots d–g.

We compared the performance of Scorr and Xcorr via Monte Carlo simulations by looking at two key aspects of colocalization analysis, true colocalization (not doubly colocalized) and mis-colocalization (wrongly colocalized peaks), as a function of *W*_2_ (Figure 2d–f). We generated two trajectories resembling a mixture of dim and bright particles flowing across the blue and red lasers (Figure 1b) and performed analyses at either low, medium, or high particle density (see above). We assessed the effect of varying the fraction of bright peaks (5% or 50%) on the efficiency of true colocalization. We also introduced a random 10% error on PT estimation to resemble the real situation of particles flowing in our flow analyzer (see SI, pp 2–3).^11^

Figure 2a–c shows the comparison of Δ*T* distributions obtained from high-density particles (1:1 mixture of bright and dim particles; see SI for results on low- and medium-density scenarios) colocalized either via Xcorr or Scorr. Overall, colocalization via Scorr allowed for a narrower Δ*T* distribution compared to Xcorr, yielding 45.3% and 69.5% of peaks colocalized within ±0–0.25 and ±0–0.5 ms, respectively, whereas Xcorr yielded only 35.2% and 57.3% of peaks colocalized within the same ranges. Fine shifting introduced an additional 10% increase in peaks colocalized within ±0–0.5 ms (Figure 2b and c, shown to compare the *fine* shift with *fine* and *coarse* shifts together, used in Scorr).

Furthermore, setting the first cutoff window (*W*_1_) to lower values greatly improved the accuracy of peaks shifting for high-density particles (Figure 2c), yielding 58.1% and 84.6% of peaks colocalized within ±0–0.25 and ±0–0.5 ms, respectively. An important consideration is that the closer the peaks are after shifting, the narrower the *W*_2_ is that can be used without a loss in colocalization. Moreover, when analyzing hundreds of particles per second, a narrow *W*_2_ is preferred to avoid errant multicolocalization of peaks.

The lower accuracy obtained using Xcorr is demonstrated by the loss of colocalized peaks at narrower cutoff windows (*W*_2_, Figure 2d). For example, Xcorr yielded a 30% loss in colocalized peaks for *W*_2_ = 4 ms (at low density) versus 0.7% when using Scorr. Scorr yielded a stable colocalization efficiency down to *W*_2_ = 1 ms, with a loss in colocalization of only ∼2% for both low and medium particles density. Below this value of *W*_2_, the efficiency dropped as well due to the 10% error upon *PT* estimation introduced in the simulations. At high particle density, the error on *PT* estimation, together with the increase in multicolocalization happening during coarse shifting, caused a systematic loss in peak colocalization even at *W*_2_ > 1 ms. However, this could be reduced by choosing a lower value for *W*_1_ (Figure 2e).

As shown in Figure 2f, the percentage of mis-colocalized peaks remained independent from the cutoff window (*W*_2_)—except for Xcorr performed on high particle density where it showed a slightly negative correlation in the lower range of *W*_2_- but not from *W*_1_, suggesting that mis-colocalization mostly occurs because of peaks multicolocalization during coarse shifting. Xcorr failed to prevent dim–bright cross-colocalization, yielding up to ∼5% and ∼22% of mis-colocalized peaks in the case of medium- and high-density particles, respectively (1:1 mixture of bright/dim particles and *W*_2_ = 4 ms). Instead, Scorr yielded almost no mis-colocalization (≤ ∼0.6% of dim peaks colocalized with bright ones) except for high-density particles. However, the percentage of mis-colocalized peaks dropped to nearly zero when setting *W*_1_ = 2 ms (Figure 2g). Thus, Xcorr loses colocalized peaks and forces the discarding of a greater percentage of total particles.

### Comparison of Scorr and Xcorr for Experimental Multicolor Bead Colocalization Analysis

**Figure 3:**
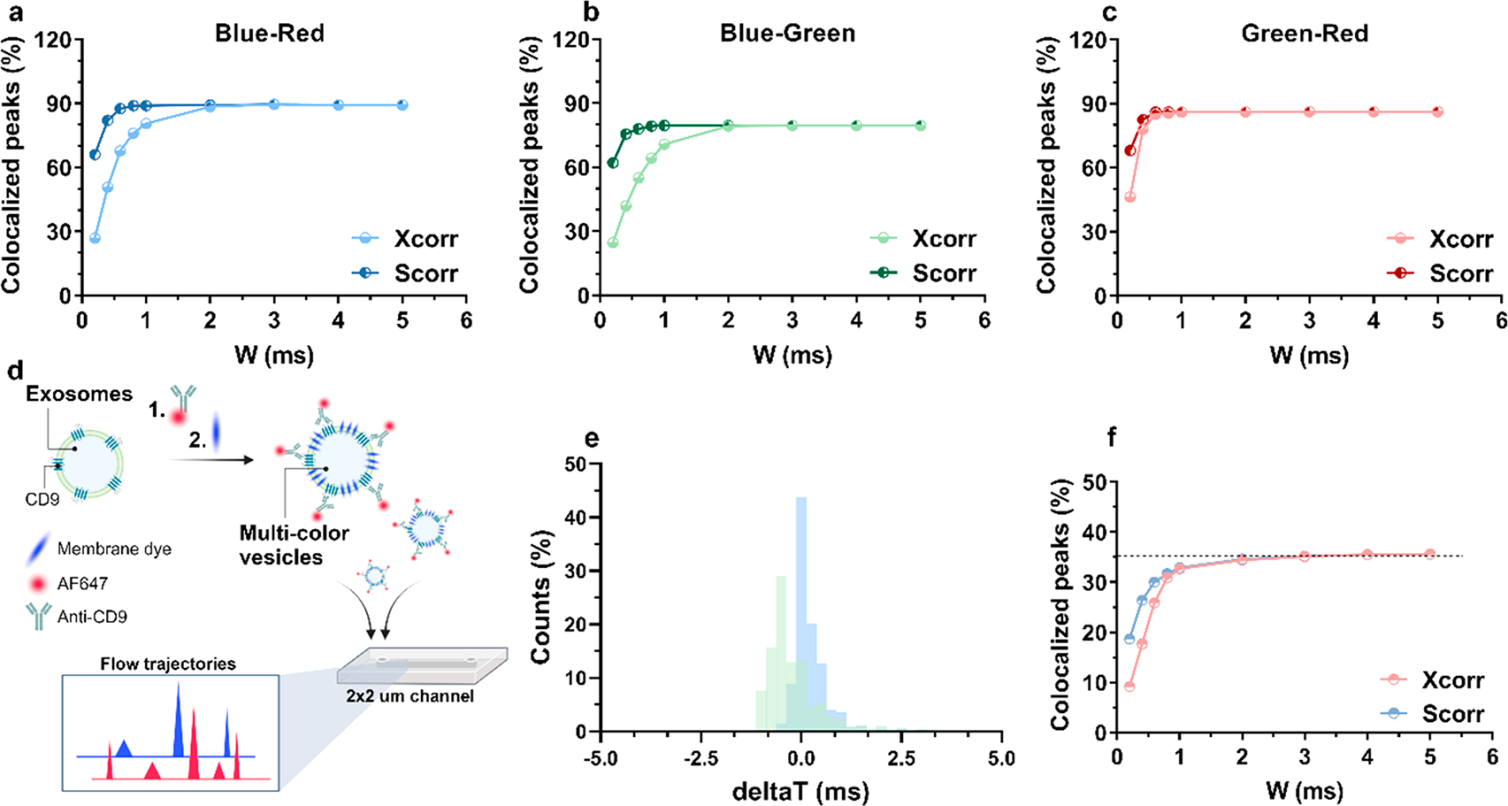
(a–c) Experimental percentage of true colocalized peaks as a function of *W_2_* (indicated as W in graphs a–c and e–f), obtained from multicolor beads analyzed at low density, for different pairwise colocalization: (a) blue–red channels, (b) blue–green channels, and (c) green–red channels. (d) Scheme for the multicolor staining of exosomes with a membrane dye and the AF647-tagged Anti-CD9 antibody. (e) Distribution of Δ*T* |*t*′_488_ – *t*_488_|, with *t*′_488_ calculated according to eqs 3 and 4 obtained from exosomes after shifting using Xcorr (green bars) or Scorr with both coarse and fine shifts (blue bars). (f) Percentage of true colocalized exosomes as a function of *W*_2_, using Xcorr (pink line) or Scorr (blue line). *W*_1_ was set to 2 ms for experiments on both multicolor beads and exosomes.

Next, we applied the two strategies for colocalization to the experimental analysis of 100 nm multicolor beads analyzed with a flow analyzer. We recorded fluorescence signals from beads in three channels—blue (488 nm), green (561 nm), and red (640 nm)—and examined the percentage of true colocalized peaks as a function of *W*_2_ (setting *W*_1_ = 2 ms) for low-density (∼4 particles/s, Figure 3a–c), medium-density (∼28 particles/s), and high-density (∼190 particles/s) multicolor beads (Figure S6 for medium- and high-density particles cases). Scorr yielded a better pairwise colocalization than Xcorr for all three trajectories (Figure 3a–c). Already at *W*_2_ = 1 ms, Scorr reduced the loss in true colocalized peaks from ∼10% to ∼0.5% for blue–red and blue–green pairs, whereas the colocalization efficiency remained the same for the green–red trajectories. The superiority of Scorr in colocalizing peaks appears even clearer for smaller *W*_2_ values; even at *W*_2_ = 0.2 ms, the loss in true colocalized peaks remained within 20%–25% when using our strategy versus 45%–70% when using Xcorr. Notably, although the initial total colocalization percentage is different for each trajectory (due to preselection of peaks during particles speed estimation, which might lead to peaks rejection if they do not present a Gaussian-like shape because, for example, of interaction with channel walls or coelution with other vesicles),^11^ the results are consistent across the three channels, demonstrating that Scorr can be used in systems with multiplexing capability.

### Single-Vesicle Colocalization Analysis on Exosomes

We assessed the performance of our strategy for particles colocalization on a real biological sample, namely, semen exosomes (∼70 nm in diameter),^11^ and compared the results from Scorr with Xcorr analysis. Thus, exosomes were incubated with Di-8-ANEPPS—a membrane intercalating dye that stains all vesicles—and an AF647-tagged Anti-CD9 antibody for two-color colocalization (Figure 3d). In agreement with results on multicolor beads, the Scorr method yielded a narrower Δ*T* distribution from colocalized peaks compared to Xcorr (Figure 3e). For instance, 73% and 31% of peaks were colocalized already within 0–0.5 ms range for Scorr and Xcorr, respectively. Although the percentage of colocalized peaks remained constant (∼35%) down to *W*_2_ = 1 ms for both Xcorr and Scorr, the latter ensured an ∼2-fold increase in colocalization for values of *W*_2_ lower than 1 ms. These results highlight the advantage of using Scorr when performing immunophenotyping of vesicles since lower values of *W*_2_ will allow for better accuracy in colocalization, even at higher vesicle densities.

## Conclusion

Here, we describe a cross-correlation method, Scorr, for use in performing multicolor colocalization in single vesicle/particle flow cytometry, which is an alternative to the classical cross-correlation approach, Xcorr. Unlike Xcorr, Scorr performs single-vesicle colocalization by exploiting information on individual particles’ transit times across the laser excitation beam width. By doing so, Scorr greatly improves both the efficiency and accuracy of colocalization analysis (higher percentage of colocalized peaks and negligible mis-colocalization). Furthermore, Scorr ensures higher reproducibility of results because it is insensitive to variables such as particle density, fraction of impurities (e.g., dye aggregates), and changes in flow rate during acquisition.

## Supporting Information Available

Derivations of eqs 2 and 3, estimation of parameters for Scorr analysis (*PTi* and ΔXi), description of the Scorr algorithm and workflow, Δ*T* distributions for low- and medium-density simulated beads, percentage of colocalized peaks as a function of *W* for high-density beads with 5% fraction of bright/dim peaks, percentage of colocalized peaks as a function of *W* obtained from high-density multicolor beads, and in-depth description of trajectory generation in simulated data (PDF)
