# Supplementary material for: Improving Multicolor Colocalization in Single-Vesicle Flow Cytometry with Vesicle Transit Time
Source: Anal Chem. 2023 Jul 5;95(28):10492–7. doi: 10.1021/acs.analchem.3c01197 (PMC10357400; doi:10.1021/acs.analchem.3c01197)
Supplement: Supplementary file 1 — ac3c01197_si_001.pdf [file ac3c01197_si_001.pdf]

Improving Multicolor Colocalization in Single-Vesicle Flow Cytometry with Vesicle Transit Time

Luca A. Andronico, *‡ Seung-Ryoung Jung,† Bryant S. Fujimoto† and Daniel T. Chiu*†

†Department of Chemistry, University of Washington, Seattle, Washington 98195, USA.

‡Department of Women's and Children's health (KBH), Karolinska Institutet, Solna 17177, Sweden.

*Correspondence and requests for materials should be addressed to L.A.A or D.T.C. (email: luca.andronico@ki.se; chiu@uw.edu).

Table of Contents

1) Monte Carlo simulation; pp. 2-3

2) Algorithm workflow for Scorr colocalization; pp. 4-5

3) Derivation of equations for *coarse* and *fine* shifting; pp. 5-6

4) Estimation of parameters in Eq. S1 and S3-S8 and vesicle transit time across the laser beam width; pp. 6

5) DeltaT distributions for low- and medium-density simulated beads; pp. 7

6) Mis-colocalization for simulated beads (5% of bright-over-dim peaks); pp. 8

7) True colocalized peaks for medium- and high-density multicolor beads; pp. 9-10

1) Monte Carlo simulation

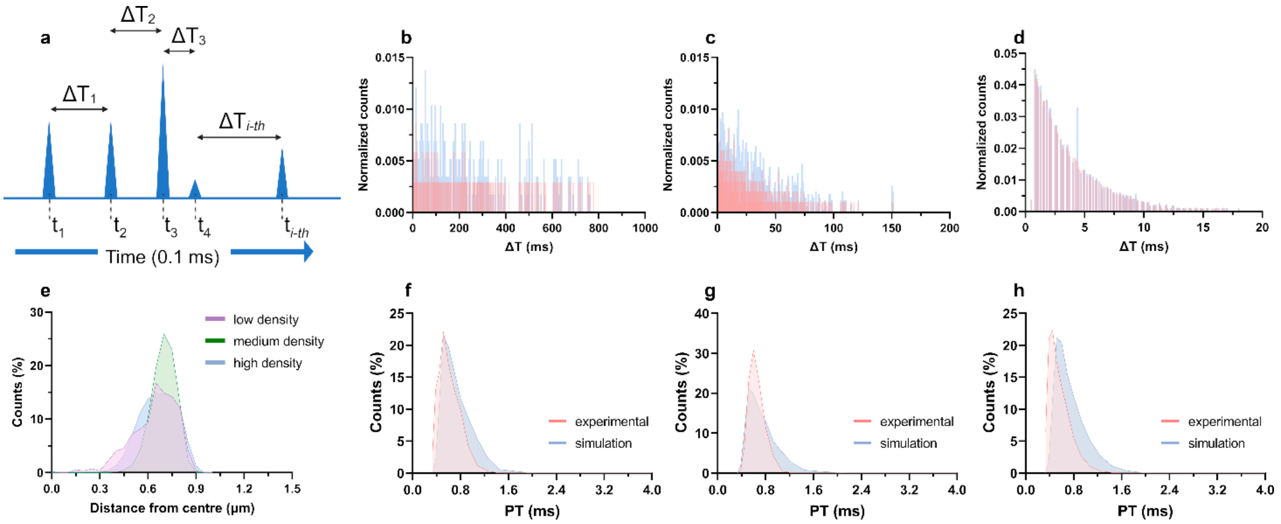

Figure S1. a, Scheme of flow trajectory in the blue channel. **b-d**, Distributions of time distances between consecutive peaks (ΔT) in the blue trajectory, obtained from experimental and simulation data on low (**b**), medium (**c**) and high (**d**) density particles. Red bars refer to experimental data (*i.e.*, multi-color beads) whereas blue bars refer to simulation results. **e**, Experimental spatial distributions of multi-color beads flowing within the 2x2 μm channel. **f-h**, Distributions of passing times (*PT*) at low (**f**), medium (**g**) and high (**h**) particles density.

The flow trajectory of channel #1 was generated as follows:

i) From experimental data on multi-color beads, we calculcated the distribution of distances between consecutive peaks at different particles densities (see Fig. S1a). We used these distributions as weights during random selection of peak occurrences in the blue trajectory. This allowed as to spread a pre-defined number of peaks (580, 3400 or 30200, for the low, medium or high density scenario, respectively) along the flow trajectory in a way more representative for the real scanarios (see Fig. S1b-c).

ii) Locations of brighter peaks (either 5% or 50% of total peak, with 3-fold higher intensity) were selected randomly, to resemble the real case of particles of different sizes flowing within the microfluidic channel.

iii) From experimental data on multi-color beads, we derived the spatial distribution of particles within the 2x2 μm channel, by rearranging Poiseuille's equation (see Eq. S1).

$$r = \sqrt{\frac{\left(\frac{1}{PT_{min}} - \frac{1}{PT_i}\right)R^2}{\frac{1}{PT_{min}}}} \qquad \text{Eq. S1}$$

R refers to the channel radius in μm, PT_{min} refers to the smallest passing time of particles (*i.e.*, fastest particle recorded) whereas PT_i refers to the passing time of the *i-th* particle. Our results (see Fig. 1e) were in agreement with the literature,[1] which shows a focusing of particles at ~ 0.6 times the channel size due to the balancing between two forces: a shear gradient (from the centre of the channel to the wall) and a lift force (wall effect) from the wall to the centre. Thus, we used the experimental distribution of particles' location as weights to

randomly assign a a given travelling speed to individual peaks. This ensured similar *PT* distributions between experimental and simulation data (see Fig S1f-h).

The flow trajectory of channel #2 was generated as follows:

i) each peak from channel #1 was shifted forward in time according to Eq. S5, S8 (see below), using the pre-seleceted parameters for laser widths and laser-to-laser distance (see main text) and the pre-assigned peak-specific travelling speed (*i.e.*, transit time).

ii) To each peak-specific travelling speed (*PT*), a random ±10% error was added, to resemble the real case of particles flowing within our µm-sized channel (see below).[1]

2) Algorithm workflow for Scorr colocalization

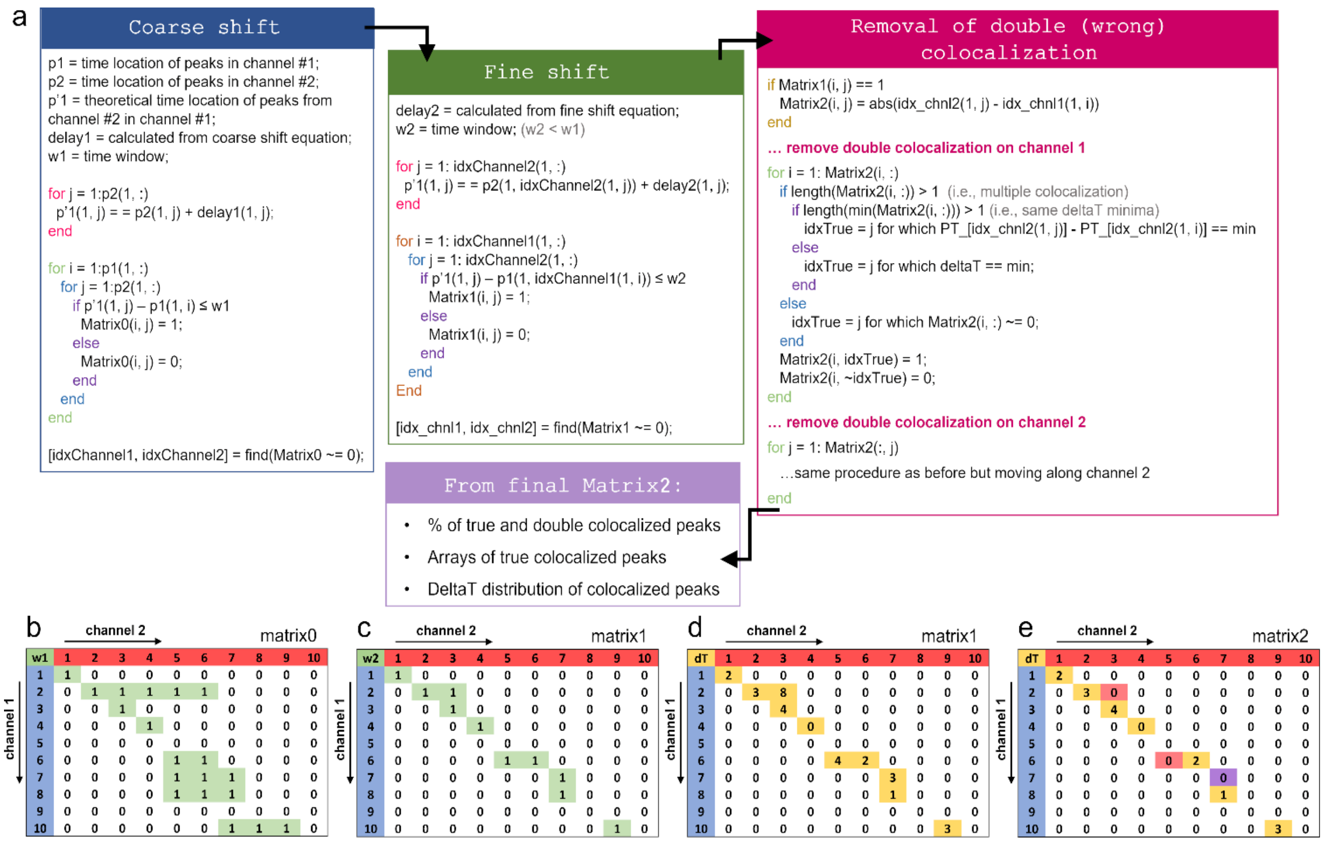

Figure S2. a, Schematic of the algorithm workflow. **b-e**, matrices generated during single vesicle-based colocalization.

Let's consider the simple case of particles flowing in a 2-color system, where laser #2 is placed after laser #1, along the flow direction. Therefore, two distinct flow trajectories (*T1* and *T2*) with same number of peaks (*N*) but different locations in time (*p1* or *p2*, with peaks in *T2* being delayed in time compared to their respective peaks in *T1*) will be measured. To achieve single-vesicle colocalization, the algorithm performs a 4-step process (see Fig. S2a):

(i) a "coarse" shift of peaks in *T1* forward in time which generates new peak locations (*p'1*), to identify initial colocalized particles according to a user-defined time window (*w1*). Specifically, *p'1* refers to the theoretical location which a peak in trajectory #1 would have in trajectory #2 according to its travelling speed.

(ii) a "fine" shift of peaks in *T1* forward in time which generates a new set of "refined" peak locations (*p'1*), to re-colocalized particles according to a user-defined time window (*w2*).

(iii) removal of any residual double colocalization of peaks in *T1* with peaks in *T2*.

(iv) removal of any residual double colocalization of peaks in *T2* with peaks in *T1*.

In each stage, the algorithm generates an NxN matrix where peaks in *T1* and *T2* run along rows or columns, respectively. After coarse shift, elements in the matrix (Matrix0, see Fig. S2b) are either 0 or 1, depending on whether the two peaks are closer (1) or farther (0) in time than *w1*. Matrix1 (see Fig. S2c) is generated after fine shift and is built in the same way as Matrix0 but considering *w2*, instead. Then, 1s are replaced by the

corresponding *p2-p'1* values (see Fig. S2d), and the algorithm proceeds with removing double colocalization on both channels (see Fig. S2e). In case of multiple colocalization, the algorithm will consider as "true" colocalized those peaks which show the smallest *p2-p'1* value (*i.e.*, which are closer in time), or (for same *p2-p'1* values) those which show the smallest change in travelling speed between *T1* and *T2* (*i.e.*, particles that are flowing in the same laminar sheet).

3) Derivation of equations for coarse and fine shifting

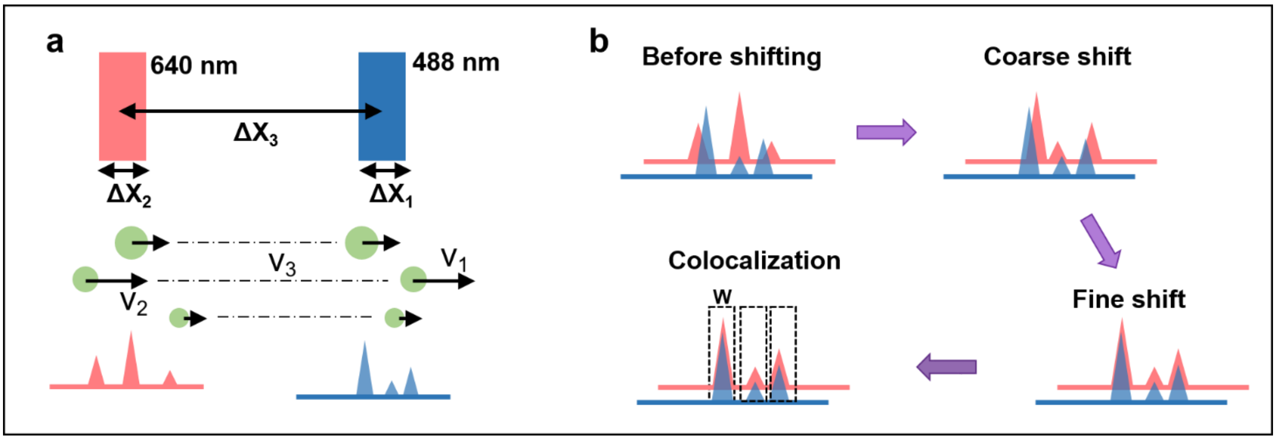

Figure S3. a, Schematic of vesicles flowing within the μm-sized channel. The green circles represent vesicles of different sizes which flow at different speeds (black arrow) across the red and blue lasers. Each peak height is matched with a corresponding size. **b,** Effect of the shifting applied to individual peaks according to Eq. S4-S7, during colocalization analysis *via* Scorr.

ΔX_1, ΔX_2 and ΔX_3 represent the blue (488nm) laser width, the red (640nm) laser width and the distance between lasers, whereas PT_1, PT_2 and PT_3 the times required to cover those respective lengths (see Fig. S3a). The travelling speed for each *j-th* particle is calculated as $v_i = \Delta X_i / PT_i$ where *i* refers to the specific time-distance pair selected (*i.e.*, *i* = 1 refers to the beam width and PT for the blue laser *etc.*).

<u>Coarse shift</u>: relies on a first assumption (HP_1) that each i-*th* vesicle—travelling along the microfluidic channel at its own speed (v_i)—does not change its velocity (for instance, due to lateral diffusion). Therefore, according to HP1, v_i remains constant throughout the channel length and we can write:

$$v_3 = v_2 \qquad \text{Eq. S2}$$

$$\frac{\Delta X_3}{PT_3} = \frac{\Delta X_2}{PT_2} \qquad \text{Eq. S3}$$

$$PT_3 = t'_{488} - t_{640} \qquad \text{Eq. S4}$$

where t_{640} is the time at which the n-*th* particle crosses the red laser and t'_{488} is the theoretical time at which the same particle should cross the blue lasers, if travelling with constant speed *v* (*i.e.*, if HP_1 holds true). Solving Eq. S3 for PT_3 and combining it with Eq. S4 we obtain an equation (see Eq. S5) which allows to calculate t'_{488} for each individual vesicle (see Fig. S3b).

$$t'_{488} = t_{640} + \frac{PT_2 \Delta X_3}{\Delta X_2} \qquad \text{Eq. S5}$$

Fine shift: Instead, if we consider that the travelling speed of each vesicle might slightly change while crossing the two lasers, we can assume (HP_2) that the velocity calculated from the different occurrence in time between peaks in the two trajectories (PT_3) and the laser-to-laser distance (ΔX_3) is an average of the two velocities estimated from the crossing time at the two lasers (PT_1 and PT_2). Therefore, we can rewrite Eq. S2 and S3 as following:

$$v_3 = \frac{v_2 + v_1}{2} \qquad \text{Eq. S6}$$

$$\frac{\Delta X_3}{PT_3} = \frac{1}{2}\left(\frac{\Delta X_2}{PT_2} + \frac{\Delta X_1}{PT_1}\right) \qquad \text{Eq. S7}$$

Solving Eq. S7 for PT_3 and combining it with Eq. S4, we obtain an equation to perform fine shifting on individual peaks (see Fig. S3b):

$$t'_{488} = t_{640} + \frac{2\Delta X_3 PT_1 PT_2}{\Delta X_1 PT_2 + \Delta X_2 PT_1} \qquad \text{Eq. S8}$$

4) Estimation of parameters in Eq. S1 and S3-S8 and vesicle transit time across the laser beam width

The parameters used in Eq. S4-S7 (*i.e.*, the laser beam widths and the pairwise distances) where estimate from a picture of the three lasers used in our setup, which was taken by using a CCD camera, and by performing line profile in ImageJ. The transit times of vesicles (*i.e.*, PT_i) across the laser beam width were estimated as previously reported.[2] Briefly, each peak was fit to a gaussian-like curve, and PTi was calculated as six times the peak width (σ). Instead, the error on PTi was calculated from the confidence intervals, namely six times the 95% confidence intervals of σ, obtained from the fitting.

5) DeltaT distributions for low- and medium-density simulated beads

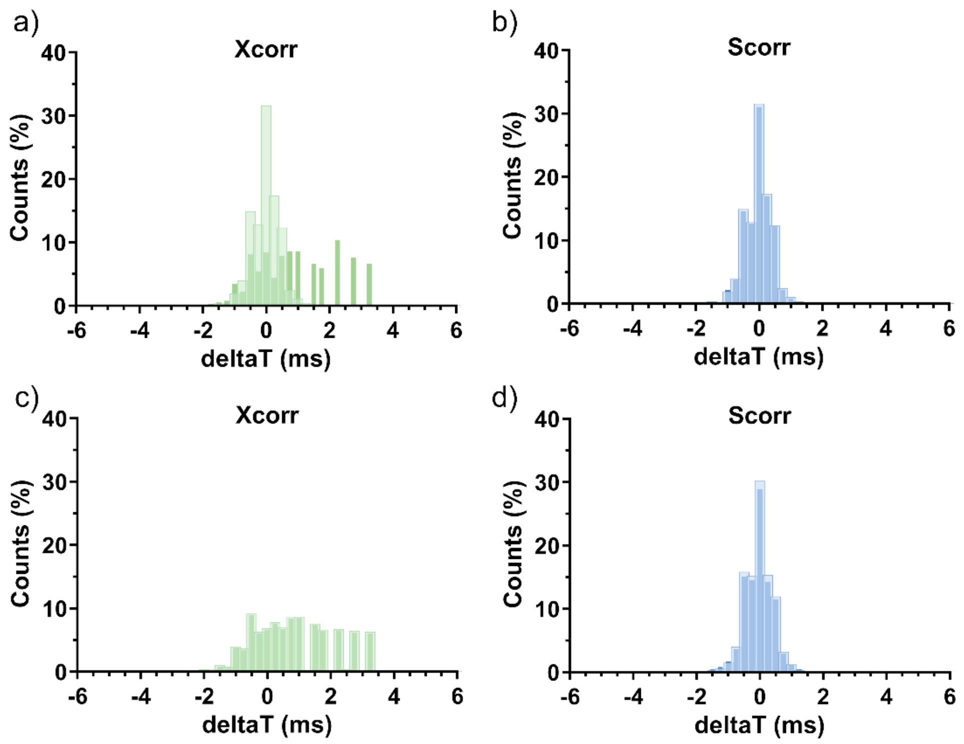

Figure S4. a-b, Distribution of deltaT |t'$_{488}$ – t_{488}| of colocalized peaks with t'$_{488}$ calculated according to Eq. 3-4 (see main text), obtained from low (**a-b**) or medium (**c-d**) simulated data (1:1 dim-bright mixture), setting W_1 equal to either 2 ms (wide bars) or 5 ms (thin bars) and W_2 = 4 ms. DeltaT are obtained after shifting using Xcorr (**a-c**) or Scorr, (**b-d**).

Similar to the case of high-density multicolor beads (see main text), colocalization *via* Scorr function greatly improved the accuracy of colocalized peaks (*i.e.*, narrower deltaT distributions). For both low- and medium-density beads, Scorr yielded ~61% and 89% of peaks colocalized within ± 0.25 ms and ± 0.5 ms, respectively. Xcorr led similar results only for low density particles, whereas values for colocalized peaks dropped to only ~21% and 37 % within the same range, for medium density particles.

6) Mis-colocalization for simulated beads (5% of bright-over-dim peaks)

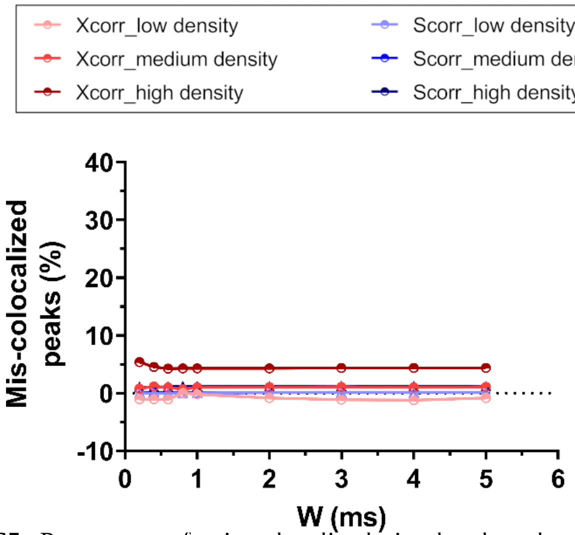

Figure S5. Percentage of mis-colocalized simulated peaks (*i.e.*, dim peaks with brighter ones) as a function of W_2 (W_1 = 2 ms), at different particles densities (see figure legend) and with 5% fraction of bright-over-dim peaks. Each line refers to shifting *via* either Xcorr (light-to-dark red lines) or Scorr (light-to-dark blue lines).

Similar to the scenario of 50% fraction of bright-over-dim peaks, Scorr ensured the false colocalizations remained low (~1%) even at high-density particles. Instead, Xcorr failed to prevent mis-colocalization, yielding up to ~5-fold higher false colocalization for the case of high-density particles (dark red curve).

7) True colocalized peaks for medium- and high-density multicolor beads

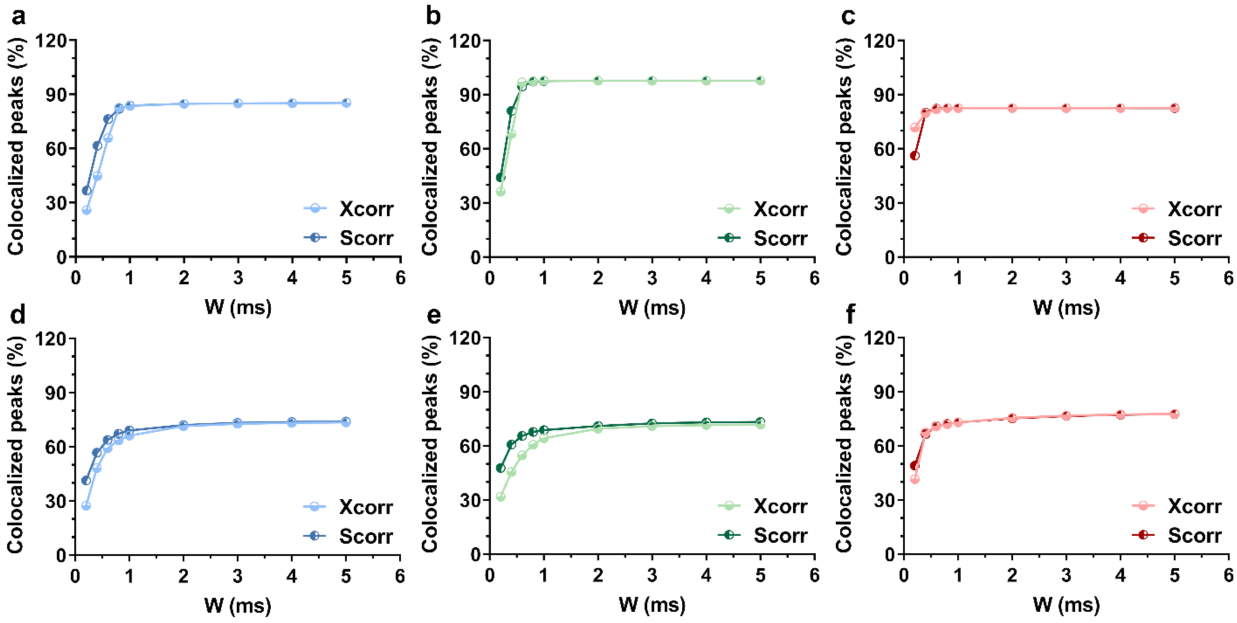

Figure S6. Percentage of true colocalized peaks as a function of W_2, obtained from multicolour beads analyzed at either medium- (**a-c**) or high-density (**d-f**). Plots refer to different pairwise colocalization: blue-red channels (**a**, **d**), blue-green channels (**b**, **e**) and green-red channels (**c**, **f**).

For the case of medium- and high-density beads, the differences in percentage of true colocalized peaks between Xcorr and Scorr are smaller than the case of low-density beads and appear only at $W_2 < 1$ ms. This might be due to an increase of co-elution events happening at higher particles density, as suggested in Fig. S7 (where the distribution of peaks intensities shifted towards ~40% higher values with increase in particles density).

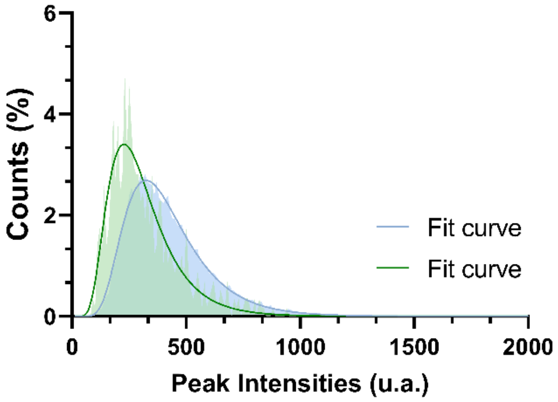

Figure S7. Distribution of peak intensities for multi-color beads at low (green curve) and high (blue curve) particles density. Solid lines represent the respective lognormal fitting.

If two particles co-elute across the laser beam, they will appear as one particle with travelling speed (*i.e.*, *PT*) somewhere in between the actual values. In a multi-color setup, a pair of particles might co-elute only when crossing one of the lasers, therefore affecting the correct estimation of particles' travelling speeds. However,

this would be a limitation of the flow setup rather than the Scorr method for colocalization, and could potentially be avoided, for instance, by adjusting the particles density together with the flow rate (to maintain high counts/sec) or introducing additives to reduce particle-to-particle interactions (more likely at high particles density) *etc*.

---

[1] Di Carlo, D. *Lab Chip* **2009**, 9, 3038-3046.
[2] Andronico, L. A.; Jiang, Y.; Jung, S. R.; Fujimoto, B. S.; Vojtech, L.; Chiu, D. T. *Anal. Chem.* **2021**, 93, 5897–5905.
